# Leaf Energy Balance Requires Mitochondrial Respiration and Export of Chloroplast NADPH in the Light^1^

**DOI:** 10.1104/pp.19.00624

**Published:** 2019-06-18

**Authors:** Sanu Shameer, R. George Ratcliffe, Lee J. Sweetlove

**Affiliations:** Department of Plant Sciences, University of Oxford, Oxford OX1 3RB, United Kingdom

## Abstract

Key aspects of leaf mitochondrial metabolism in the light remain unresolved. For example, there is debate about the relative importance of exporting reducing equivalents from mitochondria for the peroxisomal steps of photorespiration versus oxidation of NADH to generate ATP by oxidative phosphorylation. Here, we address this and explore energetic coupling between organelles in the light using a diel flux balance analysis model. The model included more than 600 reactions of central metabolism with full stoichiometric accounting of energy production and consumption. Different scenarios of energy availability (light intensity) and demand (source leaf versus a growing leaf) were considered, and the model was constrained by the nonlinear relationship between light and CO_2_ assimilation rate. The analysis demonstrated that the chloroplast can theoretically generate sufficient ATP to satisfy the energy requirements of the rest of the cell in addition to its own. However, this requires unrealistic high light use efficiency and, in practice, the availability of chloroplast-derived ATP is limited by chloroplast energy dissipation systems, such as nonphotochemical quenching, and the capacity of the chloroplast ATP export shuttles. Given these limitations, substantial mitochondrial ATP synthesis is required to fulfill cytosolic ATP requirements, with only minimal, or zero, export of mitochondrial reducing equivalents. The analysis also revealed the importance of exporting reducing equivalents from chloroplasts to sustain photorespiration. Hence, the chloroplast malate valve and triose phosphate-3-phosphoglycerate shuttle are predicted to have important metabolic roles, in addition to their more commonly discussed contribution to the avoidance of photooxidative stress.

## Abstract (precis)

Metabolic modeling reveals why mitochondrial respiration and chloroplast NAD(P)H export are required in illuminated leaves.

The role of mitochondria in leaves in the light has long been a matter of debate (Nunes-Nesi et al., 2008; Dahal et al., 2017; Tcherkez et al., 2017; O’Leary et al., 2019). This is in part because photosynthesis dominates the energetics of a leaf during the day, but also because the biochemistry of leaf mitochondria during the day departs substantially from the conventional tricarboxylic acid cycle-coupled-to-oxidative-phosphorylation mode that is the norm for nonphotosynthetic cells (Sweetlove et al., 2010; Millar et al., 2011; Tcherkez et al., 2012; O’Leary et al., 2019). Flux through the complete set of reactions of the tricarboxylic acid cycle is largely absent in the light due to allosteric inhibition of the pyruvate dehydrogenase enzyme (Tovar-Méndez et al., 2003). Instead, in C_3_ leaves, the main source of NADH within leaf mitochondria during the day is from the oxidation of photorespiratory Gly by the enzyme Gly decarboxylase. Within the photorespiratory cycle, stoichiometrically equal amounts of NADH are generated by mitochondrial Gly decarboxylase and consumed by peroxisomal hydroxypyruvate reductase (Bauwe et al., 2010). This had led to the suggestion that all of the mitochondrial NADH generated by Gly decarboxylase would be transferred to the peroxisome using a malate-oxaloacetate (OAA) metabolite shuttle system (Fig. 1). Consistent with this, studies of Arabidopsis (*Arabidopsis thaliana*) knockout mutants of the mitochondrial isoforms of malate dehydrogenase show that they have pronounced growth defects under low-CO_2_ conditions that promote photorespiration (Tomaz et al., 2010; Lindén et al., 2016). However, the stoichiometric equivalence within the photorespiratory cycle in terms of the respective production and consumption of NADH in the mitochondrion and peroxisome belies the complexity of the process by which reducing power is balanced across the metabolic system, including across subcellular compartments. Indeed, within the photorespiratory cycle itself, additional reducing power is required in the chloroplast to reassimilate released ammonium.

**Figure 1.**
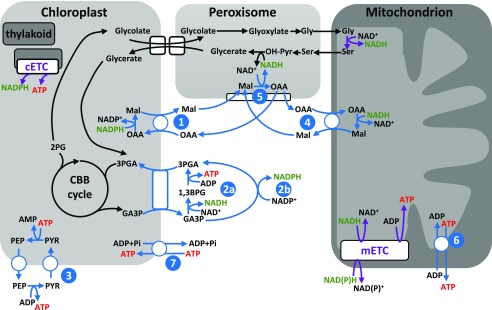
Schematic showing the principal routes by which ATP and NAD(P)H can be generated and moved between organelles in the leaf model. Purple lines indicate sites of generation. Blue lines indicate mechanisms to move ATP and/or NAD(P)H between organelles and the cytosol. Each is numbered as follows: 1, chloroplast malate valve to shuttle NAD(P)H from chloroplast to the cytosol; 2a, triose phosphate-3-phosphoglycerate (TP-3PGA) shuttle to export NADH and ATP from chloroplast to the cytosol (using cytosolic phosphorylating glyceraldehyde 3-phosphate dehydrogenase and phosphoglycerate kinase [PGK]); 2b, TP-3PGA shuttle to export NADPH from chloroplast to the cytosol (using cytosolic nonphosphorylating glyceraldehyde 3-phosphate dehydrogenase); 3, phospho*enol*pyruvate (PEP)-pyruvate shuttle to export ATP from the chloroplast to the cytosol; 4, mitochondrial malate valve to shuttle NADH from the mitochondrion to the cytosol; 5, malate-OAA shuttle to transfer cytosolic reducing equivalents as malate to the peroxisome; 6, mitochondrial adenylate nucleotide translocase to export ATP from the mitochondrion to the cytosol; 7, import of ATP into the chloroplast via the plastidial nucleotide transporter, NTT. Note that the NTT plays a role in energizing the chloroplast at night but is not thought to be important in the illuminated leaf (Flügge et al., 2011) and was therefore constrained to zero flux during the day in the FBA model. 1,3BPG, 1,3-Bisphosphoglycerate; CBB, Calvin-Benson-Bassham; cETC, chloroplast electron transport chain; GA3P, glyceraldehyde 3-phosphate; Mal, malate; mETC, mitochondrial electron transport chain; PYR, pyruvate.

The use of inhibitors of the mitochondrial ATP synthase and Gly decarboxylase suggests that a proportion of the NADH from Gly oxidation is oxidized via the mitochondrial respiratory chain to generate ATP by oxidative phosphorylation (Gardeström and Igamberdiev, 2016). For example, the addition of oligomycin to barley (*Hordeum vulgare*) protoplasts decreased the ATP-ADP ratio in both mitochondria and cytosol, whereas the chloroplast ATP-ADP ratio was unchanged (Gardeström and Wigge, 1988; Krömer and Heldt, 1991; Wigge et al., 1993). Based on experiments of this kind, it has been suggested that between 50% and 75% of the NADH from Gly oxidation is used for mitochondrial ATP synthesis (Krömer, 1995), in distinct contrast to the argument that all of the NADH is exported to maintain NADH balance within the photorespiratory cycle.

The apparent discrepancy between the Arabidopsis malate dehydrogenase mutants and the barley protoplast experiments may be explained partly by the different experimental systems used. But the actual balance between the export of mitochondrial NADH and its oxidation for mitochondrial ATP synthesis is likely to depend strongly on the overall energetic balance of the cell. Of crucial importance will be the photosynthetic photon flux density (PPFD) experienced by the leaf in relation to the flux of the Calvin-Benson-Bassham cycle and other ATP/NAD(P)H-consuming metabolism in the chloroplast. If excess energy is available, then the chloroplast can contribute to the peroxisomal demand for NADH and the cytosolic demand for ATP, exporting NADH via a malate-OAA shuttle or other dicarboxylate transporters (often referred to as the malate valve; Selinski and Scheibe, 2019) and both NADH and ATP via a TP-3PGA shuttle (Taniguchi and Miyake, 2012; Fig 1). Recent analyses of in vivo cytosolic ATP levels in Arabidopsis cotyledons using a fluorescent protein sensor are consistent with some export of chloroplast ATP even under a relatively low PPFD of 296 μmol m^−2^ s^−1^ (Voon et al., 2018). The sensor response when oligomycin was added was also consistent with the earlier barley protoplast studies, suggesting that mitochondria contribute substantially to the cytosolic ATP pool.

However, none of these experiments measure the transfer fluxes for ATP and reducing equivalents between organelles and the cytosol, and so quantitative conclusions about the relative importance of different routes cannot be drawn. Moreover, although the studies all impose slightly different light conditions, there has been no systematic investigation of the relative importance of the different routes under different energetic states. Given that the energetic balance will depend not only on the PPFD incident on the leaf, but also on the varying demands for ATP and reducing power in the different subcellular compartments, this is a complex issue. Hundreds of reactions draw on the ATP and NAD(P)H pools in the different subcellular compartments, and the net energy balance of the leaf is hence a system-level property of the metabolic network operating at steady state.

Computational models are powerful tools for understanding such system-level properties, with two main approaches being used to model metabolism, namely kinetic models and stoichiometric models. Kinetic models capture the response of enzymes, transporters, and electron transport chains to their substrates and effectors, and they provide a powerful predictive tool for analyzing the response of the system to variable conditions (Baghalian et al., 2014). For leaf metabolism, this approach has mainly focused on photosynthesis and associated processes in the chloroplast (Rohwer, 2012). However, the large number of parameters in these models and the challenge of solving the large system of ordinary differential equations generally limit the number of metabolic steps that can be included. Although recent kinetic models of photosynthesis have been expanded to include processes beyond photosynthesis, including, for example, the chloroplast costs of photorespiration, starch synthesis, and nitrate reduction (Zhu et al., 2013; Morales et al., 2018), these models do not include a complete account of the energy demands beyond the chloroplast. Therefore, these models do not accurately address the energetic interactions between the chloroplast and the rest of the cell.

In contrast, a second modeling approach, flux balance analysis (FBA), is readily scaled to incorporate the entire metabolic system of a cell (Sweetlove and Ratcliffe, 2011; Shi and Schwender, 2016; Gomes de Oliveira Dal’Molin and Nielsen, 2018). This approach simplifies the mathematical representation of the system by considering only the stoichiometry of the metabolic reactions and uses experimental constraints and an optimization objective to make predictions about flux distributions in the metabolic network at steady state (Nikoloski et al., 2015). This approach has been proven capable of making quantitatively realistic predictions of plant metabolic behavior (Basler et al., 2018; Moreira et al., 2019). A number of studies have applied FBA to C_3_ leaf metabolism (de Oliveira Dal’Molin et al., 2010, 2016; Poolman et al., 2013, 2014; Arnold and Nikoloski, 2014; Cheung et al., 2014, 2015; Lakshmanan et al., 2015), but the specific questions of the role of mitochondria in the light and the energetic coupling between subcellular compartments have not been explicitly considered.

An earlier and simplified stoichiometric model of leaf metabolism did consider the balance between mitochondrial respiration and photosynthesis (Buckley and Adams, 2011). However, that study focused specifically on respiratory CO_2_ release, and the simplicity of the model meant that the energy demands of the leaf were not fully accounted for (e.g. the costs associated with subcellular transport of metabolites and ions). Moreover, the central question that we are asking, specifically what is the fate of mitochondrial photorespiratory NADH, cannot be predictively explored in the model of Buckley and Adams (2011) because the fraction of NADH retained in the mitochondrion is a defined parameter rather than a prediction of the model. Hence, the aim of this work was to analyze a stoichiometric model of leaf metabolism using a diel FBA framework with a specific focus on metabolic and energetic interactions between chloroplasts and mitochondria in the context of the energetic balancing of the system as a whole under scenarios of different energy availability or utilization.

## RESULTS

### Modeling Framework and Setup

**Figure 2.**
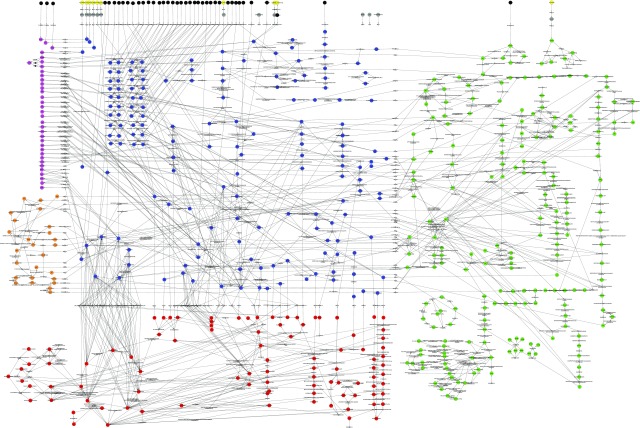
Diagrammatic representation of the metabolites and reactions present in the model. Metabolites and cofactors involved in large number of reactions (such as CO_2_, water, ATP, ADP, and inorganic phosphate) are omitted for the sake of clarity. Metabolites are represented by colored circles and reactions by gray diamonds. The metabolite circle color corresponds to its subcellular localization (blue, cytosol; red, mitochondrion; green, plastid; orange, peroxisome; purple, vacuole; gray, apoplast; yellow, environment; black represents model outputs). In this bipartite graph, the nodes are reactions and metabolites and the edges are reaction-metabolite associations. Reaction and metabolite names follow the conventions used in the PlantCyc metabolic pathways database (Schläpfer et al., 2017).

**Figure 3.**
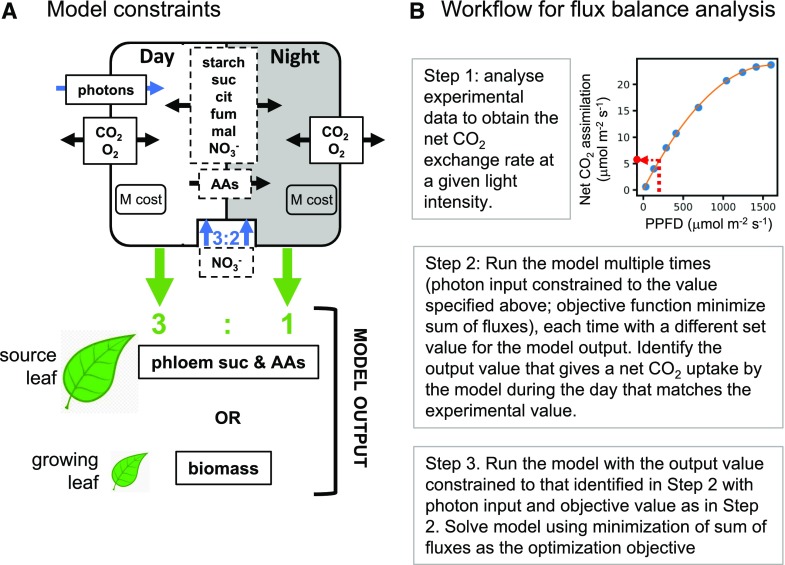
Schematic showing the setup and simulation procedure for a leaf diel FBA metabolic model. A, Constraints that were applied to the model. Blue arrows indicate input constraints. Green arrows indicate output constraints. Metabolites in the dashed box are those that can be stored in one phase of the model (day or night) and passed to the other for utilization. B, Procedure used to simulate leaf metabolism accounting for the nonlinear relationship between photosynthetic rate and light intensity. Data shown were extracted from Donahue et al. (1997). AAs, Amino acids; cit, citrate; fum, fumarate; M, maintenance; mal, malate.

The engagement of mitochondrial ATP synthesis in a leaf in the light is likely to depend upon the balance between the available light energy (PPFD) and energy demand. To assess this, we set up a diel FBA model of primary metabolism of an Arabidopsis leaf (Shameer et al., 2018) to operate at different light intensities. The charge- and proton-balanced model accounted for all reactions required for autotrophic synthesis of the main components of biomass (cell wall, lipid, protein, and nucleotides). As a flux balance model, total production of energy (ATP) and reducing power (NADH) must be balanced by their consumption. This will include the multitude of reactions and intracellular transport steps required to generate a defined metabolic output, as well as maintenance costs. The model contains full stochiometric descriptions of the electron transport chains of chloroplasts and mitochondria, including alternative modes such as cyclic photophosphorylation and uncoupled mitochondrial respiration (although these alternative modes were not constrained to specific values). A diagrammatic representation of the model is shown in Figure 2. The figure is also available in its original Cytoscape (Shannon et al., 2003) format as Supplemental Figure S1, and this format is fully searchable by metabolite or reaction name. The basic configuration of the model accounting for the day and night phases of leaf metabolism and the constraints applied are shown in Figure 3A. The model was free to choose from a range of storage compounds that can be accumulated in either day or night and then released in the complementary temporal phase. Two types of leaf were considered and were constrained by defining the nature of the metabolic output: first, a mature source leaf, where the output of the model was export of sugars and amino acids to the phloem in relative proportions found in Arabidopsis phloem sap (Wilkinson and Douglas, 2003); and second, a growing leaf, where the output of the model was synthesis of biomass components for growth of new leaf tissue as defined by experimental measurements and described in the AraGEM model (Gomes de Oliveira Dal’Molin et al., 2015).

To capture the nonlinear response of photosynthesis to light, the model was constrained to experimental measurements of the relationship between PPFD and net CO_2_ assimilation rate for Arabidopsis (Donahue et al., 1997). This was achieved by varying the metabolic output of the model until the net CO_2_-uptake flux predicted by the model matched the experimental net CO_2_ assimilation rate (Fig. 3B). To account for light lost by reflectance and transmission, the amount of light available to the model was set to be 90% of the PPFD (Zhu et al., 2010). The objective function of the FBA optimization problem was to minimize the sum of all fluxes. The fluxes in an FBA solution are not necessarily uniquely defined, and this limitation was addressed by performing flux variability analysis (Mahadevan and Schilling, 2003). Conclusions have only been drawn where the flux variability analysis range was less than 10% of the flux value. All code and associated model files, including all constraints applied to the model, are available at https://github.com/ljs1002/Shameer-et-al-Role-of-mitochondria-in-C3-leaf-during-the-day.

### Experimental Rates of Photosynthesis Are Achievable in Diel FBA Models of Both Source and Growing Leaves

**Figure 4.**
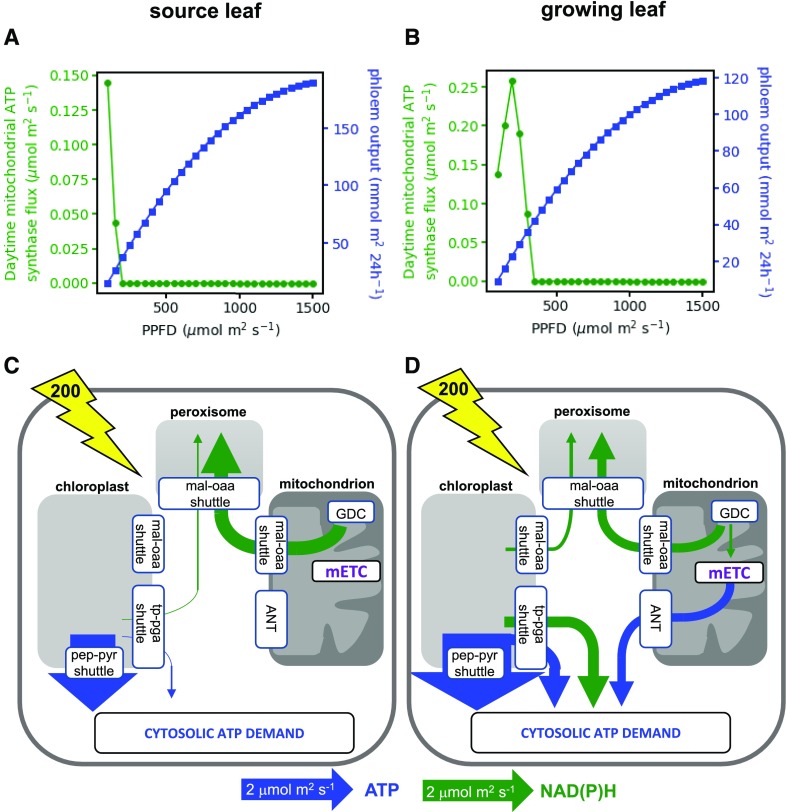
Daytime energy shuttling between organelles in models of source and growing leaves. A and C, Source leaf. B and D, Growing leaf. A and B, Flux of mitochondrial ATP synthase (green) and diel model output (blue) at a range of light intensities (PPFD). C and D, Schematic of key fluxes transferring ATP or NAD(P)H between subcellular compartments at a PPFD of 200 μmol m^−2^ s^−1^ (indicated by the yellow lightning bolt arrow). The widths of the arrows are scaled relative to the flux as indicated under the diagram. Blue arrows are fluxes that involve ATP, and green arrows are those that involve NAD(P)H. For more details of the metabolite shuttles, see Figure 2. Abbreviations are as in Figure 2; ANT, adenine nucleotide translocase; GDC, Gly decarboxylase.

Surprisingly, in both the source and growing leaf models, initial simulations revealed that it is possible to achieve the experimentally constrained net CO_2_ assimilation rate without any flux through the mitochondrial electron transport chain or mitochondrial ATP synthase. To examine the influence of the energy balance of the system on this result, we ran the model over a range of light inputs (Fig. 4, A and B). The source leaf model was able to generate its output (export of sugars and amino acids to the phloem) without requiring mitochondrial ATP synthesis as long as the light intensity was 200 μmol m^−2^ s^−1^ or greater (Fig. 4A). For the growing leaf, a higher light intensity of 400 μmol m^−2^ s^−1^ was required before mitochondrial ATP synthesis was dispensable (Fig. 4B). The higher light intensity is understandable because the biosynthesis of biomass components requires more energy than the synthesis of sugars and amino acid for phloem export (as can be seen from the lower molar output of biomass per unit of light compared with that of sugars and amino acids; compare Fig. 4, A and B). In the growing leaf model at light intensities where daytime mitochondrial ATP synthesis was required, there was a complex relationship between its flux and light intensity that is difficult to intuitively rationalize but that likely has its origins in the interaction between a linear increase in energy (light) input and a nonlinear increase in CO_2_ assimilation and model output. The results demonstrate that, provided sufficient light energy is available, daytime mitochondrial ATP synthesis is not necessarily required in either source or growing leaves.

### In the Absence of Mitochondrial ATP Synthesis, High Rates of Export of ATP from the Chloroplast Are Required to Satisfy the Demand for Cytosolic ATP

To examine how the model meets the demand for cytosolic ATP in the absence of mitochondrial ATP synthesis, we inspected the fluxes of the organellar metabolite shuttles (Fig. 1) in the source leaf model with a light input of 200 μmol m^−2^ s^−1^ (Fig. 4C). All predicted fluxes and flux variability ranges from this model are shown in Supplemental Data Set S1. It can be seen that even at this low light intensity, there is excess energy in the chloroplast such that the majority of the cytosolic ATP demand of a source leaf can be met from exported chloroplast ATP (Fig. 4C). In this simulation, the export of ATP occurs mainly by the PEP-pyruvate shuttle with a much smaller export of ATP and NADPH via the TP-3PGA shuttle. The peroxisomal requirement for NADH is met from the NADH generated by mitochondrial Gly decarboxylase, with the NADH transferred to the peroxisome by the action of mitochondrial and peroxisomal malate dehydrogenase and malate-OAA shuttles. As a result, there is no NADH available for mitochondrial ATP synthesis. In contrast, in the model of a growing leaf at the same light intensity (Fig. 4D), a portion of the mitochondrial NADH from Gly decarboxylase is used for mitochondrial ATP synthesis. Because this reduces the amount of mitochondrial NADH that can be transferred to the peroxisome, the chloroplast malate valve becomes operational to export NADPH (as malate) from the chloroplast to enable the peroxisomal NADH requirement to be met, with the chloroplast meeting 21% of the peroxisomal NADH demand. This demonstrates the flexible and interdependent nature of the energy exchanges between organelles and how these are dependent upon the overall energy status of the system.

### The Capacity of the Chloroplast ATP Shuttles Dictates the Use of Mitochondrial Respiration to Meet Cytosolic ATP Demands

Although it is known that the chloroplast can use metabolite shuttles to export ATP, especially in conditions of energy excess (Gardeström and Igamberdiev, 2016), the metabolite shuttles that allow export of chloroplastic ATP may lack the capacity to support the ATP export fluxes shown in Figure 4. We therefore constrained the upper limit of the shuttles transporting ATP according to experimental estimates of the maximal catalytic activities of the relevant enzymes. The chloroplast PEP-pyruvate shuttle requires the activity of pyruvate orthophosphate dikinase to convert pyruvate to PEP in the chloroplast (Fig. 1). This enzyme generally has a low activity in C_3_ leaves, and in Arabidopsis leaves, a value of 0.9 ± 0.2 μmol mg^−1^ chlorophyll h^−1^ has been reported for the maximal catalytic activity (Ishimaru et al., 1997). This converts to 0.034 μmol m^−2^ s^−1^, which was set as the upper limit for this reaction flux in the model (for details of the conversion factors used, see “Materials and Methods”). Similarly, data for the maximal catalytic activities of phosphorylating NAD-GAPDH (93 μmol m^−2^ s^−1^; Gibon et al., 2004) and nonphosphorylating NADP-GAPDH (0.33 μmol m^−2^ s^−1^; Rius et al., 2006) in Arabidopsis leaves were used to constrain the upper limit of the chloroplast TP-3PGA shuttle.

**Figure 5.**
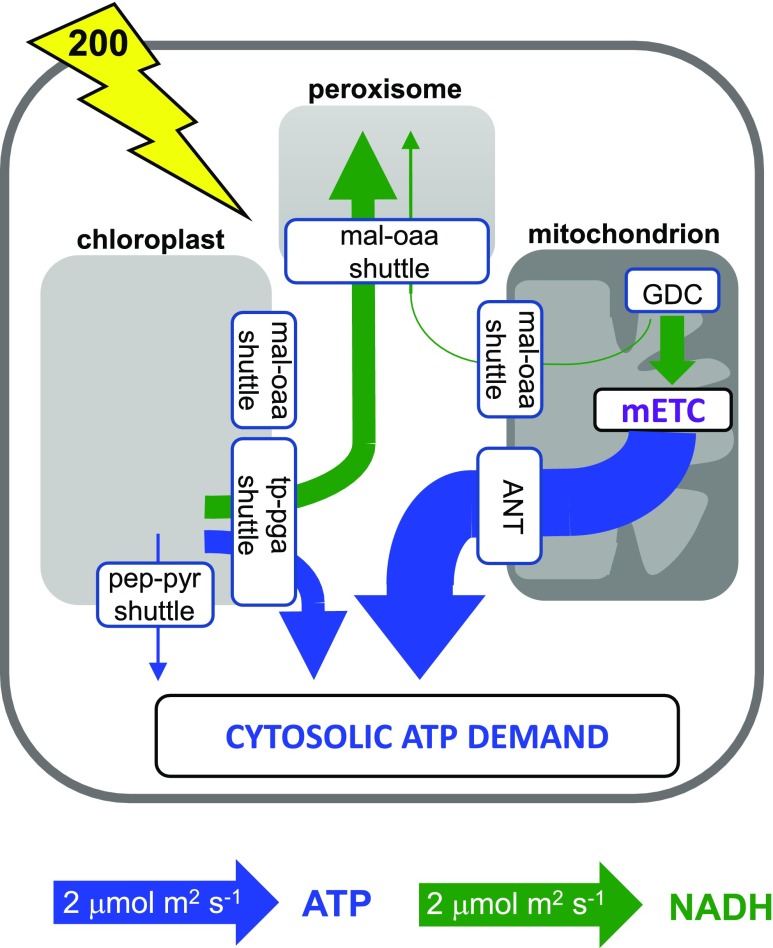
Daytime energy shuttling between organelles in a model of a source leaf at a PPFD of 200 μmol m^−2^ s^−1^, with the upper bounds of the chloroplast ATP exporting metabolite shuttles constrained to experimental values of the relevant enzyme maximum catalytic activities. Features of the schematic are as in Figure 4, and abbreviations are as in Figures 2 and 4.

When these constraints were applied to a source leaf model with an incident PPFD of 200 μmol m^−2^ s^−1^, the predicted pattern of ATP and NADPH exchanges between organelles changed markedly compared with those shown in Figure 4. The full set of predicted fluxes is shown in Supplemental Data Set S2, and key fluxes are illustrated schematically in Figure 5. The chloroplast PEP-pyruvate shuttle now shows negligible flux, a result that is consistent with experimental observations. For example, analysis of transgenic tobacco (*Nicotiana tabacum*) with reduced cytosolic pyruvate kinase showed that the main role of this enzyme in leaves is associated with nocturnal respiration (Grodzinski et al., 1999). Moreover, isotope labeling experiments in cocklebur (*Xanthium strumarium*) leaves suggest that cytosolic PEP-pyruvate interconversion in the light is in the wrong direction for chloroplast ATP export (Tcherkez et al., 2011). As an alternative to the chloroplast PEP-pyruvate shuttle, the constrained model uses the chloroplast TP-3PGA shuttle to export excess chloroplast ATP to the cytosol (Fig. 5). However, because this shuttle also leads to stoichiometric export of NAD(P)H, there is a limit to the amount of ATP that can be exported in this way in the balanced system. In the constrained model, the flux through the TP-3PGA shuttle is sufficient to provide the majority of the NADH required in the peroxisome (Fig. 5). However, because this flux provides insufficient ATP to meet the cytosolic demand, a substantial flux through mitochondrial ATP synthase was predicted (Fig. 5). Note that in this model solution, the cytosolic phosphorylating NADP-GAPDH reaction carries zero flux. This reaction would allow the export of ATP and NAD(P)H by the chloroplast TP-3PGA shuttle to be partially uncoupled, causing the ratio of NAD(P)H to ATP exported to be increased. However, in the scenario being modeled, the requirement is for more ATP than NAD(P)H to be exported, hence the nonphosphorylating NADP-GAPDH reaction, which generates only NADPH and carries no flux. Moreover, the maximum rate of the reaction from experimental data (0.33 μmol m^−2^ s^−1^) means that it cannot have a major bearing on the overall exchange of energy and reducing power between compartments in this system. The conclusion from this analysis is that capacity limits in the chloroplast ATP-exporting system lead to the use of the mitochondrial respiratory chain to generate sufficient ATP to meet cytosolic demands in a source leaf. This conclusion also holds for a growing leaf (Supplemental Data Set S2).

To assess the validity of this conclusion, we looked at experimental data in which components of the chloroplast TP-3PGA shuttle had been genetically manipulated. The three components are the triose phosphate translocase (TPT), cytosolic GAPDH, and cytosolic PGK. Of these, manipulations of the TPT are not particularly informative, because the TPT is also the principal route of carbon export from the chloroplast and reduction in TPT leads to a major compensatory alteration in carbon flows between starch and Suc as a result (Heineke et al., 1994; Häusler et al., 1998; Walters et al., 2004). However, mutant and transgenic plants in which the amounts of the other two components have been suppressed have less dramatic phenotypes and provide a more suitable basis on which to assess the validity of the model prediction. Based on the results from the model, we predict that reduction in the capacity of the chloroplast TP-3PGA shuttle would restrict the supply of ATP to the cytosol. Furthermore, we suggest that this is unlikely to be compensated by increased mitochondrial ATP synthesis because the model shows that when ATP export from the chloroplast is constrained, almost all of the available intramitochondrial NADH (from Gly decarboxylase) is already being used for ATP synthesis (Fig. 4). The only way that mitochondrial ATP synthesis could be further increased would be to import additional reductant into the mitochondria. In the light, this is unlikely to occur to any significant extent via pyruvate import because of inhibition of pyruvate dehydrogenase (Tovar-Méndez et al., 2003) and is also unlikely via import of carboxylic acids such as malate or citrate because of the redox poise of mitochondria in relation to the cytosol (Igamberdiev and Gardeström, 2003). Hence, we would expect that suppression of cytosolic GAPDH or PGK would lead to reduced cytosolic (and possibly total cellular) ATP levels and reduced rates of Suc synthesis. The latter might manifest as lower daytime Suc levels and possibly slower growth but could also be compensated for by increased starch accumulation and increased Suc export at night.

Consistent with this, Arabidopsis lines with decreased cytosolic GAPDH are slow growing and have decreased levels of ATP in illuminated leaves (Rius et al., 2006). These lines also showed decreased leaf respiration, which is not consistent with our prediction. However, respiration rates in leaves can only be measured in the dark, and the measured rates are probably more reflective of nocturnal metabolic modes where GADPH will be required to supply mitochondria with respiratory substrate as pyruvate via glycolysis. Measurements in the GAPDH mutant lines were made 15 min after the light was switched off, and this is sufficient time for the chloroplast ATP supply to be depleted and the ATP export capacity of the chloroplast to become irrelevant. Results from Arabidopsis knockouts of the PGK3 gene encoding cytosolic PGK are also consistent with our predictions: these lines had reduced growth but also reduced leaf Suc and higher starch levels than the wild type (Rosa-Téllez et al., 2018).

Ultimately, the most direct test of the model predictions about the extent of mitochondrial ATP synthesis in the light would be to measure the rate of respiration in illuminated leaves. Unfortunately, there is no current method to do this, due to the complication of photosynthetic gas exchange occurring simultaneously with respiratory gas exchange.

### Parsimonious Use of Light Energy May Explain the Use of Mitochondrial Respiration during the Day

Given that the chloroplast has an excess of energy, even at low light levels (Fig. 4), the question arises as to why Arabidopsis leaves do not invest in a greater capacity of the chloroplast ATP exporting metabolite shuttles to allow excess chloroplast ATP to be utilized in the cytosol. We have already shown that the extent to which cytosolic ATP demand is met by the chloroplast and mitochondrion is dependent on the overall energy balance of the system (Fig. 4). We therefore looked again at how we deal with the variable light input into the model. The only reactions in the model that can use the incoming photons are PSI and PSII in the chloroplast. Hence, according to the FBA problem, the sum of the fluxes of PSI and PSII must equal the incident PPFD. In the source leaf model when PPFD is 200 μmol m^−2^ s^−1^, this leads to an excess of both ATP and NADPH being produced in the chloroplast in relation to the constrained amount of CO_2_ being fixed. Some of this excess energy is exported from the chloroplast, as shown in Figures 4 and 5, while the rest is dissipated using metabolic cycles (Cheung et al., 2015; Supplemental Data Sets S1 and S2).

In practice, there are also nonphotochemical quenching (NPQ) mechanisms that function to dissipate excess light energy before it reaches the photosystems (Müller et al., 2001). NPQ functions to prevent photooxidative stress (Li et al., 2009) and is activated by feedbacks from the chloroplast reduction state and ATP demand (Ruban et al., 2012). In effect, NPQ serves to balance the photosynthetic production of NADPH and ATP with the energy demand of the chloroplast. If the balance is perfect, there will be little excess chloroplast NADPH or ATP, and the rest of the metabolic system may therefore be required to be parsimonious with its use of reducing power and energy.

To assess this, we changed the way that we set the light input into the model. Rather than forcing all of the incident light to be used for photosynthesis, we instead constrained the model so that the incident PPFD represented an upper bound on the summed PSII and PSI fluxes, leaving the model to use less light if this better satisfied the optimization objective function. Given that the optimization objective is to minimize the sum of all fluxes to achieve a set metabolic output flux and the utilization of light fluxes will contribute significantly to this flux sum, the model will likely use less light than the maximum PPFD, in order to match photosynthetic ATP and NADPH production rates with the total demand of the system for energy and reducing power. This indirectly mimics the operation of NPQ. The full set of flux predictions for this simulation is provided in Supplemental Data Set S3. Note that in this simulation, no constraints were placed on the chloroplast ATP-export shuttles and, hence, apart from light utilization, it is comparable to that of Figure 4 and Supplemental Data Set S1.

**Figure 6.**
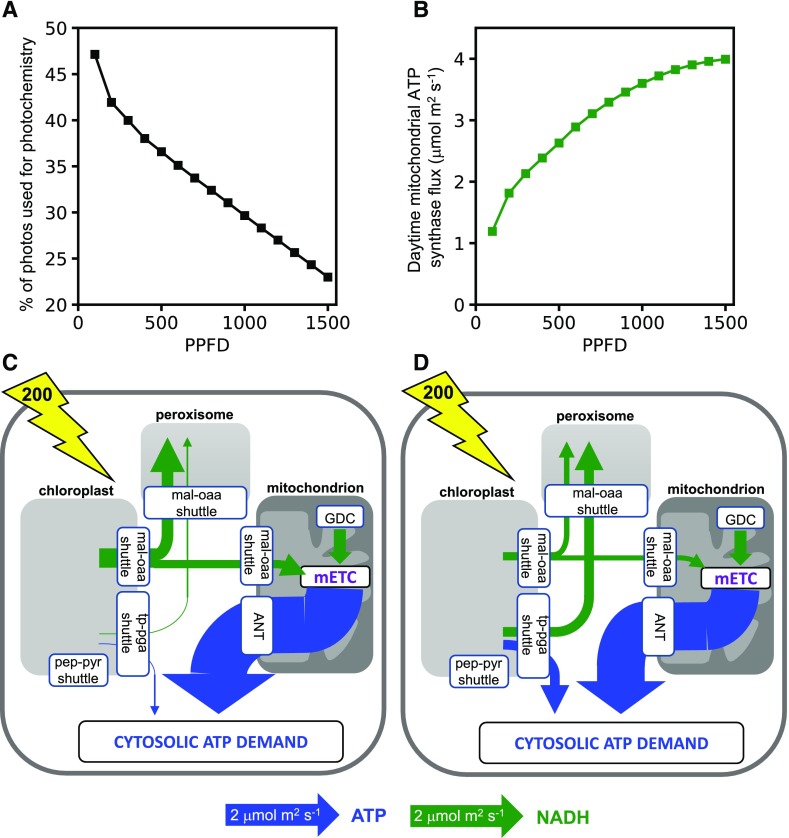
Daytime energy shuttling between organelles in a model of a source leaf in which light utilization by the chloroplast photosystems is permitted to be less than the incident PPFD. A, The photons used by the photosystems at a range of incident PPFD values. B, Daytime mitochondrial ATP synthase flux at a range of incident PPFD values. C, Schematic of the principal fluxes transferring ATP or NAD(P)H between subcellular compartments at an incident PPFD of 200 μmol m^−2^ s^−1^ and no constraints on chloroplast metabolite shuttles. D, As in C but with a constraint of 0.75 μmol m^−2^ s^−1^ on the maximum flux of the chloroplast malate-OAA shuttle (Fridlyand et al., 1998). Features of the schematic are as in Figure 4, and abbreviations are as in Figures 2 and 4.

As can be seen in Figure 6A, only a fraction of the incident light is required by the photosystems in order to meet the net CO_2_ assimilation constraint, with 42% of the incident light being used at a PPFD of 200 μmol m^−2^ s^−1^, falling to 23% at a PPFD of 1,500 μmol m^−2^ s^−1^. These predictions are broadly in line with experimental measurements for Arabidopsis (Kalituho et al., 2006; Khanal et al., 2017). Note that the light used in these simulations was not necessarily the theoretical minimum. Comparison of the light used against that obtained when the model was rerun with minimization of light use as the objective function revealed that slightly greater than the minimal amount of light was used (1% to 4% greater) at PPFDs of greater than 200 μmol m^−2^ s^−1^ (Supplemental Table S1). Interestingly, above 200 μmol m^−2^ s^−1^, the model began to use cyclic electron transport in the chloroplast, with the fraction of cyclic electron transport relative to linear electron transport rising from 4% to 11% as light increased (Supplemental Table S2). Mitochondrial ATP synthesis was required throughout the light range (Fig. 6B), suggesting that this is important to achieve the minimal sum of fluxes and low light use while meeting the CO_2_-uptake and model-output constraints. To test this, the same simulation was run with mitochondrial ATP synthesis constrained to zero flux. This resulted in a light utilization rate by the photosystems of 94.7 μmol m^−2^ s^−1^, 13% more than the 83.9 μmol m^−2^ s^−1^ used when mitochondrial ATP synthesis was operative. Hence, the operation of mitochondrial ATP synthesis to satisfy the cytosolic ATP demand results in a more energy-use-efficient state of the leaf metabolic system.

### Export of Energy of Reducing Equivalents and ATP from the Chloroplast Is Predicted Even at Low Light Intensities

The fluxes obtained from the model at an incident PPFD of 200 μmol m^−2^ s^−1^ but with no constraint on the minimum amount of light used and no constraints on mitochondrial ATP synthesis or any of the intercompartmental ATP/reducing equivalent shuttles revealed that even when only a small proportion of the incident photons are being utilized by the photosystems, there is still some export of reducing power and ATP from the chloroplast (Fig. 6C). The flux through the chloroplast malate valve was dominant in this scenario, providing reducing equivalents in the form of malate to the peroxisome and to the mitochondrion, the latter to support a high rate of mitochondrial ATP synthesis (Fig. 6C). A smaller export of ATP and reducing power from the chloroplast occurred via the TP-3PGA shuttle (Fig. 6C), and there was no flux through the chloroplast PEP-pyruvate shuttle. Both chloroplast ATP-exporting shuttles were operating below experimentally estimated maximal capacities (see “The Capacity of the Chloroplast ATP Shuttles Dictates the Use of Mitochondrial Respiration to Meet Cytosolic ATP Demands” section). However, the flux of the chloroplast malate valve (1.81 μmol m^−2^ s^−1^) exceeded the upper flux limit of 0.75 μmol m^−2^ s^−1^ estimated by kinetic modeling (Fridlyand et al., 1998). When the upper bound of the chloroplast malate-OAA transporter in our model was constrained to this lower value, additional flux through the TP-3PGA shuttle was observed (Fig. 6D; although still below the experimental maximum value) to provide the balance of reducing equivalents for the peroxisome and the balance of the cytosolic ATP demand.

The fact that the export of chloroplast ATP and reducing equivalents occurred even when light utilization was being minimized by the model suggests that this energy export is necessary to enable the experimentally constrained amount of carbon fixed to be converted to sugars and amino acids and exported to the phloem. This is consistent with the observation that the export of chloroplast ATP and reducing equivalents occurs in all of the various simulations we have described, including that of a growing leaf. Export of chloroplast ATP is dispensable, with the model still able to generate a feasible solution when all chloroplast ATP shuttles were constrained to zero flux (Supplemental Data Set S4). This is unsurprising given the relatively small ATP export flux shown in Figure 6C. However, if all possible routes of NAD(P)H export from the chloroplast were blocked in the model, then the model displayed lower light use efficiency, using 48% of the incident 200 μmol m^−2^ s^−1^ PPFD compared with 42% without the NAD(P)H chloroplast export constraint (Supplemental Data Set S5). This was because the model was forced to use a proportion of fixed photoassimilate as a respiratory substrate in the mitochondrion: starch accumulation was increased by 5% to allow high rates of vacuolar citrate storage at night (approximately 5-fold higher), which was then transferred to the mitochondrion during the day, when the action of isocitrate dehydrogenase generated NADH for respiration. The predicted rates of citrate accumulation, one order of magnitude lower than the rate of starch accumulation (moles of citrate per night as moles of hexose equivalents per day), are unrealistically high.

## DISCUSSION

### The Role of Mitochondria in the Light

The modeling analysis described here provides a system-level view of energy balancing across the leaf metabolic network and between subcellular compartments. As such, it provides insight into the integrated role of mitochondria in leaves in the light. Given the capacity limits on the various shuttles that permit the export of reducing equivalents and ATP from the chloroplast, the analysis predicts that mitochondrial ATP synthesis allows the leaf to meet the demand for ATP in the cytosol (Fig. 5). This conclusion applies to both source leaves and to growing leaves that are actively synthesizing new biomass. As a consequence, the majority of the mitochondrial NADH generated by Gly oxidation is used for ATP biosynthesis by oxidative phosphorylation, and only a relatively minor proportion is exported from the mitochondrion as malate to supply the NADH for the peroxisomal reactions of the photorespiratory pathway. Hence, our analysis suggests that the main function of leaf mitochondria during the day is to use photorespiratory Gly as a respiratory substrate to generate ATP, with only a minor role in supplying peroxisomal NADH. This contrasts with conclusions drawn from experimental analyses of Arabidopsis mutants deficient in one or both isoforms of mitochondrial malate dehydrogenase (required to generate mitochondrial malate for export), where a combination of strong reduction in growth exacerbated by low CO_2_ and an alteration in abundance and labeling of Gly and Ser at low CO_2_ emphasized the role of mitochondria in supplying reducing equivalents for the peroxisome to sustain photorespiration (Tomaz et al., 2010; Lindén et al., 2016). However, the experimental studies did not quantify the rate of mitochondrial malate export, and so conclusions about the magnitude of this flux relative to the rate of mitochondrial Gly oxidation and oxidative phosphorylation are not possible. It is important to note that the predicted flux of mitochondrial malate export in our model when chloroplast ATP export was constrained was not zero (Fig. 5), and even a small imbalance in the photorespiratory pathway can lead to rapid photorespiratory metabolite accumulation that is likely to inhibit growth and metabolism (Timm et al., 2016). Our prediction that mitochondrial NADH from Gly oxidation is predominantly used for ATP synthesis corroborates experimental studies using respiratory inhibitors (Gardeström and Igamberdiev, 2016) and in vivo fluorescent sensors (Voon et al., 2018).

### Flexible Energetic Coupling between Organelles

The flux maps of Figures 4–6 show rather different flux modes responsible for providing energy and reducing equivalents to the cytosol and peroxisome. In these flux modes, the chloroplast and mitochondria make markedly different contributions, and different shuttle systems are used to export organellar ATP and/or reducing equivalents to the cytosol. Yet in each of these scenarios, the same rate of CO_2_ assimilation and the same rate of export of sugars and amino acids to the phloem were achieved (Supplemental Data Sets S1–S4). This demonstrates that these different flux modes are equivalent in terms of carbon use efficiency. Hence, it is stoichiometrically possible for mitochondrial respiration in the light to be entirely dispensed with, although this requires higher rates of export of ATP from the chloroplast than is likely possible in the plant, given capacity limits of the chloroplast ATP export shuttles.

### The Leaf Has More Energy than It Needs, Even at Low Light Intensities

One of the more surprising observations that emerged from this quantitative analysis of energy utilization in leaves is that even at a low incident light intensity of 200 μmol m^−2^ s^−1^ there is substantially more energy available than is required for the leaf to generate experimentally observed rates of CO_2_ assimilation and to utilize the fixed carbon for the synthesis of Suc and amino acids for export to the phloem. Our analysis shows that this is connected to the use of efficient methods of energy metabolism within the model: in simulations in which minimal amounts of light were used, the model used mitochondrial ATP synthesis to meet cytosolic ATP demands (Fig. 6). The contribution of mitochondrial respiration to high energy use efficiency can be explained by the superior stoichiometry of the process, yielding approximately twice as much ATP per photon compared with linear electron transport in the chloroplast (Kramer and Evans, 2011). As a result, at a PPFD of 200 μmol m^−2^ s^−1^, the model needs less than half the available light energy to be absorbed by the photosystems. Indeed, in real leaves, much of the incident light is dissipated before it reaches the photosystems by NPQ, as is apparent from experimental measurements in a range of plants (Demmig-Adams et al., 2008; Khanal et al., 2017). Typically, the fraction of light energy dissipated by NPQ increases as PPFD increases, reflected in our model by a decrease in the fraction of light used as PPFD increases (Fig. 6). Consequently, much of the focus has been on the photoprotective role of NPQ at high light. Less attention has been paid to the fact that NPQ continues even at low light (Demmig-Adams et al., 2008; Khanal et al., 2017). It has been suggested that NPQ is necessary because of sink limitation (i.e. the capacity of the system to utilize photochemical energy for growth of sink tissues is less than the energy produced if 100% of the incident light is used for photosynthesis; Adams et al., 2013). Our analysis suggests that this is not the whole story, because even if all of the experimentally constrained assimilated carbon is used for export of sugars and amino acids to the phloem, the model does not require 100% of the incident light energy. From this work, it is not possible to say whether the use of energy-efficient mitochondrial respiration drives the dissipation of more light energy by NPQ or vice versa. But the apparent importance of metabolic energy efficiency in our models, even when light energy is in excess, may explain the impact of transgenic introduction of more energy-efficient bypasses of photorespiration on overall plant productivity (South et al., 2019).

### The Metabolic Importance of Export of Chloroplast Reducing Equivalents

Whereas mitochondrial respiration is stoichiometrically dispensable with little impact on the flux distribution, the same is not true for chloroplast export of reducing equivalents, without which unrealistically high rates of nocturnal citrate accumulation are required to sustain mitochondrial respiration. This can be rationalized as follows: when chloroplast export of ATP is below the capacity limits of the export shuttles, mitochondrial ATP synthesis is required to meet the cytosolic ATP demand; the stoichiometrically balanced production and consumption of NADH in the photorespiratory cycle means that if NADH from mitochondrial oxidation of photorespiratory Gly is used for mitochondrial ATP synthesis, then another source of NADH must be used to sustain the peroxisomal hydroxypyruvate reductase reaction. The chloroplast is the only net source of reducing equivalents in the system. Hence, if mitochondrial ATP synthesis is active, there must be an increased rate of export of reducing equivalents from the chloroplast during the day or an increased rate of starch accumulation to provide reducing equivalents (citrate was predicted) from the night phase. The prevailing view in the literature is that export of chloroplast reducing equivalents during the day mainly occurs in conditions of excess light energy and functions to regulate the NADPH-NADP ratio in the chloroplast stroma to avoid photooxidative stress (Taniguchi and Miyake, 2012; Selinski and Scheibe, 2019). The analysis here demonstrates that export of chloroplast reducing equivalents during the day is an important component of the energy balance of the whole system and not just the chloroplast, allowing the metabolic demands of ATP in the cytosol and NADH in the peroxisome to be met in an efficient manner. This places the malate valve and other shuttle systems capable of exporting chloroplast NADPH to the cytosol as central players for achieving a balanced leaf metabolism and illustrates the degree of energetic coupling between organelles in leaves.

### Limits of the Current Approach

Although the FBA modeling presented here demonstrates how a stoichiometric model can provide insight into system-level properties of metabolic networks such as energy balancing, this type of modeling does not incorporate regulation at the enzyme/protein or metabolite level. Despite this, FBA can generate remarkably realistic flux predictions, (Williams et al., 2010; Chen et al., 2011; Cheung et al., 2013), and this is partly because regulation at the enzyme level (i.e. feedback/feedforward regulation by allosteric effectors) acts to maintain metabolite steady state, which is a core constraint of FBA. Nevertheless, some details of the flux predictions from FBA may be incorrect, as they are not captured in this indirect way. For example, the direction of flux through near-equilibrium reactions, such as malate dehydrogenase, is strongly influenced by the concentrations of the reactants and coenzymes involved. Thus, the direction of the malate-OAA shuttle that transfers reducing equivalents between the mitochondria and cytosol is dependent on the respective NADH-NAD^+^ ratios in the mitochondrial matrix and cytosol. Because of the steady-state constraint, FBA models are blind to these ratios, and such shuttles are free to run in either direction in our model. Constraints could be added on shuttle directionality, but this would require accurate data on subcellular NADH-NAD^+^ ratios in the different scenarios being measured, and this is hard information to acquire.

Another example of the influence of regulatory mechanisms is cyclic versus linear electron transport in the chloroplast. In many of the solutions presented here, photosynthetic electron transport is exclusively linear (i.e. equal fluxes of the PSII and PSI reactions), even though our model is capable of cyclic electron transport (Cheung et al., 2015). Cyclic electron transport is considered to be an important mechanism by which the ratio NAD(P)H-ATP is adjusted to match demand (Finazzi and Johnson, 2016). In practice, the fraction of cyclic electron transport is very low under nonstress conditions (Morales et al., 2018), and so this will have little quantitative impact on the simulations presented here. The one scenario in which cyclic electron transport is used in the simulations we analyzed was when incident PPFD was greater than 200 μmol m^−2^ s^−1^ in a model free to use less than the incident PPFD and with the constraints of using the metabolic output to attain an experimental rate of net CO_2_ assimilation while minimizing the sum of fluxes of the system. The complex nature of these constraints makes it difficult to intuitively understand why different energy-rebalancing methods are chosen by the model.

Another issue is that FBA tends not to choose parallel routes to achieve the same end. Hence, our model prefers to dissipate excess reducing power in the chloroplast using the xanthophyll cycle rather than using, for example, uncoupled mitochondrial respiration (Vanlerberghe et al., 2016; Dahal et al., 2017). To accurately predict the relative engagement of the variety of energy-rebalancing systems used in a leaf, one would have to capture the regulatory mechanisms that respond to system-level readouts such as energy and redox poise in different subcellular compartments. And ultimately, this is a matter of enzyme/transporter kinetics and their effect on the concentrations of ATP-ADP and NAD(P)H-NAD(P)^+^ in different subcellular compartments. For example, the rate of NADH generation by Gly oxidation in C_3_ plants can exceed the capacity at which it can be oxidized in the cytochrome branch of the respiratory chain, and this triggers NADH oxidation via the uncoupled alternative respiratory chain (Igamberdiev et al., 1997). Various kinetic models have included aspects of this regulation in the chloroplast in order to predict the engagement of cyclic electron transport, nonphotochemical quenching, and other electron sinks such as the water-water cycle, malate valve, and nitrate reduction (Riznichenko et al., 2009; Zaks et al., 2012; Matuszyńska et al., 2016, 2019). But these models lack consideration of extrachloroplastic metabolism that, as our FBA modeling demonstrates, can have a major bearing on the overall energy balance of the system and hence the engagement of energy-balancing regulatory mechanisms. Ultimately, this modeling problem might be solved by coupling kinetic models that capture essential regulatory aspects of the system with stoichiometric-FBA models that can make predictions of flux distributions in large metabolic networks. This is extremely challenging because tight coupling of the two approaches renders the FBA problem nonlinear, calling for new approaches for the analysis of more loosely coupled models, which is beyond the scope of this study.

In the shorter term, some of these aspects might be better captured indirectly in FBA models by the use of additional data-based constraints. For example, as comprehensive, quantitative proteomic data sets for plants become more widely available, it will be possible to systematically introduce capacity constraints on enzymes and transporters (when catalytic constants values are known), which may generate more precisely defined flows of energy and reductant between subcellular compartments (Davidi and Milo, 2017). Additionally, systematic accounting of reaction thermodynamics can also help (Hamilton et al., 2013), but this will only substantially improve the models if it can be combined with measurements of in vivo mass action ratios, which remains challenging in eukaryotes due to subcellular compartmentation.

## CONCLUSION

The diel FBA framework presented here allows leaf metabolic network fluxes to be predicted for different scenarios of energy availability (light intensity versus CO_2_ assimilation) and utilization (source leaf versus growing leaf). The analysis revealed that capacity limits of metabolite shuttles for the export of chloroplast ATP mean that a substantial proportion of NADH generated by photorespiratory Gly oxidation must be respired by mitochondria to generate sufficient ATP to meet cytosolic demands. This, in turn, requires that reducing equivalents are exported from the chloroplast in order to meet the peroxisomal demand for NADH to keep the photorespiratory cycle running. This analysis provides a new metabolic perspective for the role of the malate valve and other chloroplast NADPH-exporting shuttles and emphasizes the importance of mitochondrial respiration in illuminated leaves.

## MATERIALS AND METHODS

### Computational Modeling

A stoichiometric model of central metabolism, PlantCoreMetabolism_v1_2 (Shameer et al., 2018), was used. The model is available as an xml file in SBML format at https://github.com/ljs1002/Shameer-et-al-Role-of-mitochondria-in-C3-leaf-during-the-day. FBA problems were set up and solved using custom Python 2 scripts, the COBRApy package (Ebrahim et al., 2013), and the CPLEX solver. All code required to reproduce the results in this article are available as a series of Jupyter notebook files at https://github.com/ljs1002/Shameer-et-al-Role-of-mitochondria-in-C3-leaf-during-the-day. A list of constraints common to all simulations is given in Supplemental Information S1.

### Unit Conversion Factors for Experimentally Measured Enzyme Capacity Constraints

In some simulations, constraints were applied to the upper bounds of reactions that are components of the metabolite shuttles that export ATP and/or reducing equivalents from the chloroplast. Experimental data for the maximal catalytic capacity of these enzymes were available in units of nmol min^−1^ g^−1^ fresh weight or nmol min^−1^ mg^−1^ protein, in contrast to the flux unit of the model, which was μmol m^−2^ s^−1^. To convert, the following data were used: specific leaf area of Arabidopsis (*Arabidopsis thaliana*), ∼50 m^2^ kg^−1^ dry weight (i.e. 1 g dry weight = 0.05 m^2^; Weraduwage et al., 2015); Arabidopsis dry weight:fresh weight ratio = 0.088 (i.e. 1 g dry weight = 11.4 g fresh weight; Arnold and Nikoloski, 2014). Hence, 11.4 g fresh weight = 0.05 m^2^ and 1 g fresh weight = 0.0043 m^2^. Therefore, 1 nmol min^−1^ g^−1^ fresh weight = 0.00387597 mmol s^−1^ m^−2^ (1:0.0043:1,000:60). To convert from per mg of protein to g fresh weight, we used a value of 5.7 mg protein g^−1^ fresh weight of Arabidopsis rosette leaves (Strand et al., 2000).

### Supplemental Data

The following supplemental materials are available.

**Supplemental Figure S1.** Cytoscape format version of the model diagram shown in Figure 2.
**Supplemental Table S1.** Comparison of light used by the photosystems at different incident light intensities for two modeling scenarios.
**Supplemental Table S2.** Predicted relative use of linear versus cyclic electron transport in the chloroplast at different incident light intensities.
**Supplemental Information S1.** List of constraints common to all simulations.
**Supplemental Data Set S1.** Predicted fluxes and flux variability analysis in source and growing leaf diel FBA models with a light input of 200 μmol m^−2^ s^−1^ and constrained to achieve an experimentally measured CO_2_ assimilation rate.
**Supplemental Data Set S2.** Predicted fluxes and flux variability analysis in source and growing leaf diel FBA models with a light input of 200 μmol m^−2^ s^−1^ and constrained to achieve an experimentally measured CO_2_ assimilation rate plus additional constraints on the upper bounds of reactions of the chloroplast ATP-exporting TP-3PGA shuttle and PEP-pyruvate shuttle to match experimental measurements of the maximal catalytic capacity of the respective enzymes.
**Supplemental Data Set S3.** Predicted fluxes and flux variability analysis in source and growing leaf diel FBA models with a light input of 200 μmol m^−2^ s^−1^ and constrained to achieve an experimentally measured CO_2_ assimilation rate.
**Supplemental Data Set S4.** Predicted fluxes and flux variability analysis in a source leaf diel FBA model with a light input of 200 μmol m^−2^ s^−1^ and constrained to achieve an experimentally measured CO_2_ assimilation rate.
**Supplemental Data Set S5.** Predicted fluxes and flux variability analysis in a source leaf diel FBA model with a light input of 200 μmol m^−2^ s^−1^ and constrained to achieve an experimentally measured CO_2_ assimilation rate.
